# Mr. Clutterbuck, on an Ambiguous Case of Hydrocephalus

**Published:** 1799-10

**Authors:** H. Clutterbuck

**Affiliations:** Walbrook


### *To the Editors of the Medical and Physical Journal.*

GENTLEMEN,

I TRANSMIT to you the following case of Hydrocephalus, for insertion (if it meets your approbation) in your valuable Journal. My attention has been too forcibly of late called to this subject, by the loss of a darling child. There was a degree of obscurity in the symptoms in this case, which might readily lead to a false judgment of the disease, and which, in fact, did induce a medical friend, whose aid I solicited, and on whose discernment I had had a ſtrong reliance, to doubt of the real nature of the affection, for a conſiderable time after its commencement.

The diagnoſis, if not the moſt difficult, is unqueſtionably one of the moſt important parts of medical ſcience, for none has a greater influence on the event. I am willing to hope that the annexed caſe and remarks may, on ſome future occaſion, lead to the more ready detection of a diſeaſe, which, I am perſuaded, in children eſpecially, is not unfrequently overlooked.

I am, with much reſpect, yours, &c.

WALBROOK, *Sept.* 16, 1799. H. CLUTTERBUCK.

T. C. a little more than two years of age, for the laſt twelve months had enjoyed but an indifferent ſtate of health. The abdomen was large, and the ſtate of the bowels irregular. About two months preceding the preſent illneſs, he was affected with ſevere inflammation of the eyes, and which extended ſuperficially over great part of the head and face, and left a ſcabby eruption on the ſcalp, which diſcharged conſiderably. On Thurſday, the 4th of July, 1799, he was obſerved to droop, and to ſhew a diſinclination for food. The following night he ſlept leſs ſoundly than uſual, and vomited on being taken out of bed in the morning. The ſkin was hot, he was thirſty, and the tongue was covered with a whitiſh cruſt; he refuſed food, and the bowels were coſtive; the tendency to vomit was encouraged by giving a tea-ſpoonful of antimonial wine, which, at the ſame time, evacuated the bowels. On Saturday and Sunday the child continued as before, much indiſpoſed, but with no other appearances than thoſe common to children in a febrile ſtate. The diſorder was attributed to cold; and this opinion was confirmed by obſerving, that the head was inclined to the left ſhoulder, with ſome degree of rigidity, the child crying out when the neck was attempted to be ſtraitened.

July 8th. On Monday the feveriſh ſymptoms had rather increaſed; the lips were parched, and the thirſt was greater. I now firſt began to ſuſpect ſome local affection of the head, and that a foundation was laying for hydrocephalus. There was an unuſual appearance in the eyes, but which I could not well deſcribe. I thought I obſerved a tendency to ſquinting at times, but this was ſo ſmall and fleeting, as to be unnoticed by the by-ſtanders, and was readily ſuppoſed to be the imaginary creature of my fears. There was no reaſon to think the power of viſion impaired: the pupils contracted readily on expoſure to light. The bowels were ſtill coſtive. The pulſe was regular and ſtrong, and hardly quicker than in health. This ſtate of the pulſe, likewiſe, gave me ſome alarm; for it was leſs irritated than in proportion to the general diſorder of the ſyſtem.

There

There was, however, no preternatural flownefs. The child fhewed an unwillingnefs to be kept erect, was reftlefs, fretted much, with a conftant defire of changing his fituation, and of being carried from place to place. The eruption on the fcalp which before fubfifted now became dry, and ceafed to difcharge altogether.

It became now a queftion in what light to view the diforder; whether as hydrocephalus, or as fimple fever? For myfelf, I had adopted the former opinion; but it was imagined my fears had magnified the evil, and that the fymptoms were no more than thofe common to children in the teething age. It was refolved, however, not to lofe fight of the former fuppofition, and efpecially as the ftate of the pulfe warranted the ufe of fuch evacuations as might be indicated. A blifter was applied accordingly on the top of the head, and the dry fcabs were rubbed with the ointment of cantharides, to folicit a return of the difcharge. A purgative was given of fcammony and calomel, which operated readily and freely. The room in which the child was kept was darkened, and the heat of the fkin moderated by cool air and drinks.

On the 9th and 10th he continued much in the fame way, without any material change of fymptoms. His fleep at night was irregular and difturbed. The purgative was given daily, together with occafional fmall dofes of antimony, to induce a moifture on the furface of the body. He took no food during this time, but drank freely of toaft and water.

Thurfday the 11th.—The child was reftlefs the beginning of the night paft, but flept foundly towards morning. To-day he appears confiderably better; he is lefs hot, lefs fretful, and notices, with apparent fatisfaction, thofe around him. The tongue is ftill white, but lefs dry. The appearance of the eyes is natural, and the pupils contract readily. The pulfe is ftrong, and of the natural frequency *. The inappetency for food ftill continues.

Friday, 12th, morning.—The feverifh fymptoms increafed towards evening yefterday: yet the night has been paffed more quietly than before, with

* I have not thought it worth while to notice the exact number of pulsations in a minute, though they were actually measured. The frequency of the pulse is influenced by such trivial causes, and so often varies from change of posture, of respiration, &c. &c. that an actual enumeration may well be neglected, as affording no satisfactory conclusion, or guide to the judgment. Its general condition, with regard to frequency, may be ascertained with sufficient precision, without the aid of a stop-watch.

with a good deal of found fleep. The heat of the fkin is abated, and he has eaten, for the firft time thefe four days, fome bread foaked in tea, with much apparent relifh. He is unwilling to be taken out of bed. The appearance of the eyes is natural. He is obferved to keep his hand almoft conftantly applied over the right eye, which, however, with the other, has a natural appearance, and the countenance expreffes no pain, and hardly differs from health, except in being fomewhat flufhed.

I was now ready to believe I had been miftaken in my fufpicions of injury exifting in the brain. The diforder feemed about to terminate in the moft favourable manner. But a confiderable change for the worfe took place in the courfe of the day. The feverifh fymptoms increafed confiderably towards evening. He fhewed great tendency to doze, but his fleep was unquiet and difturbed, and in it he moaned frequently and fighed. The pulfe was not perceptibly altered, but there was a change in the manner of his breathing which greatly alarmed me. He breathed flowly, with long intervals, and expiration was frequently accompanied with a long-continued moan. (This fymptom I had particularly remarked fome time before, in the cafe of a young man, who had for feveral months laboured under fymptoms of oppreffed brain, and in whom I found a large accumulation of water after death.) An unufual appearance of the eyes again ftruck me; they feemed to be differently directed: but the change was fo flight, as hardly to be perceived by the attendants, to whom I pointed it out. The pupils were not perceptibly enlarged, and contracted readily to the light. He had had two or three loofe motions in the day, and as he appeared to be uneafy in his bowels, the antimonial was difcontinued.

Saturday 13th, ten in the morning. The feverifh fymptoms ran high till three in the morning. He dozed conftantly, with frequent moaning as before. At this time the fcammony with calomel was repeated: he appeared better about five o'clock, and eat heartily of bread foaked in tea as before. He flept foundly after this for the fpace of two hours, without moaning. He is now heavy and drowfy, and his breathing continues flow, with moaning and fighing. The pupils are now evidently enlarged, and contract lefs readily than they did to the light. The eyes are at times flightly diftorted. There is no tendency to delirium, nor any apparent defect of vifion. There has been no evacuation by ftool fince yefterday morning, but he makes water frequently, though not involuntarily. The fkin is hot and dry, and the face flufhed: the features are compofed, and betray no uneafinefs. The pofture in which he lies is perfectly natural and eafy. The pulfe is quicker than it was. The purgative was repeated.

Two o'clock, afternoon. Continues to doze; at times quietly, but for the moft part is reftlefs, and moans frequently; refpiration as before. Countenance exhibits more diftrefs, and the mufcular ftrength is evidently much impaired. He appears to be fenfible to furrounding objects, but the eyes look dull, though without diftortion: the pupils little, if at all, dilated. Has had two copious ftools. The pulfe is moderate in ftrength and frequency, is regular, but ftill what one would term flightly feverifh. Tongue white. The feet and legs were ordered to be immerfed in hot water, and a large blifter to be applied to the occiput, and another to the nape of the neck.

Eleven at night. The heat of the fkin is much increafed, and the pulfe and breathing are quickened. The dozing continues, but the child is evidently rendered uneafy by the blifters.

Sunday 14th, ten in the morning. He continued dozing till one in the morning, when he awoke, and took his bread and tea in a ravenous manner, the eyes appearing wild and diftorted at the time. Had a lax motion foon afterwards, and flept pretty quietly for fome hours. At this time his attention to what is paffing around him fhews him to be fenfible, and he has replied feebly to fome ordinary queftions. The pupils are large, but they contract on expofure to light. The heat of the fkin is confiderable. The pulfe is pretty ftrong, and regular, not exceeding 110 ftrokes in a minute. The blifters have difcharged freely. The breathing is now quick and regular, without moaning, as in ordinary cafes of accelerated circulation.

Monday 15th, noon. The whole of yefterday and laft night have been paffed more quietly than before. The child has dozed almoft conftantly, waking at intervals; but the fleep has been apparently more found and natural. He has taken bread foaked in tea freely, every four or five hours, when offered him, but he does not afk for it. He refufes broths, and other animal food, but takes fruit eagerly. The febrile fymptoms are more moderate than whilft the blifters were acting. The countenance is compofed. The eyes are at times flightly diftorted: the pupils are large, but contract to the light, though lefs readily than they did a day or two back, when the febrile fymptoms ran higher.

Tuefday 16th, morning. The night has been paffed in feemingly quiet fleep, waking now and then for a few minutes at a time. Pulfe regular, but quicker, and more feeble. Heat of fkin confiderable. The eyes appear more infenfible: the hearing and touch not vifibly impaired.

Evening. Had a natural motion. Continues dozing as before, but takes food at regular intervals, when offered. In all other refpects nearly as before. On being taken out of bed juft now, and held erect on the nurfe's lap, he fainted almoſt immediately; the eyes became glaſſy, with cold ſweats; and he appeared in danger of inſtant diſſolution*. Theſe ſymptoms ſoon went off on his being replaced in bed, but they left him evidently conſiderably more languid and weak than before.

Wedneſday 17th, morning. He continued dozing till one o'clock in the morning, when on offering him his uſual food, it was obſerved he had ſcarcely power to ſwallow, though he evidently wiſhed to take it. Upon the whole, the child appeared to get rapidly worſe. Slight convulſive motions of the eyes and mouth were obſerved to take place now and then; there was likewiſe ſome frothing at the mouth. The ſkin continues hot, and the face is fluſhed.

Ten at night. The pulſe grows quicker and weaker: in other reſpects much the ſame.

Thurſday 18th, morning, Stupor continues, Pulſe quick, though tolerably ſtrong. Swallows a little water occaſionally when put in his mouth with a ſpoon. Appears wholly inſenſible.

Evening. The breathing has become quicker, and is performed with labour. Pulſe very rapid and ſmall. A good deal of brown frothy ſaliva has iſſued from the mouth. The heat of the extremities keeps up.

Friday morning. About the middle of the night the breathing became more laborious, with much rattling in the throat. Pulſe nearly as before, rapid but regular, with clammy ſweats, all foreboding ſpeedy diſſolution. Some efforts to cough were made, with the effect of removing the phlegm, and rendering the breathing more eaſy.

Evening. After ſome hours the breathing became again exceedingly laborious, the pulſe too rapid and feeble to be counted, and death cloſed the ſcene about four in the afternoon, on the fifteenth day of the diſeaſe.

The

* Did this ariſe from the preſſure of the water on the baſis of the brain? Or was it ſimply faintneſs, ſuch as we obſerve to take place on ſuddenly removing a perſon greatly debilitated, from the recumbent, to the erect poſture, and which is probably owing to the veſſels being unable to tranſmit the blood in a direction to which they have for ſome time been unaccuſtomed? A few months ago I met with a caſe much in point:—A little girl recovering from a malignant ſcarlet fever, and ſtill too weak to quit her bed, was attacked with inflammation of the parotid gland, which, after three days of moſt acute pain, preventing ſleep altogether, ſuppurated largely and burſt. A few hours afterwards, on being taken ſuddenly out of bed, ſhe dropped her head, and died inſtantly in the mother's arms, without a ſtruggle, or convulſive motion of any kind.—This may ſuggeſt a caution in ſimilar circumſtances.

The equivocal nature of the fymptoms, during a confiderable part of the progrefs, made an examination after death much to be defired. For the following account I am indebted to the friendly offices of Mr. ASTLEY COOPER, Lecturer on Anatomy and Surgery, at St. Thomas's Hofpital.

*Appearances on Diffection.*

" As fome fufpicions had been entertained, that difeafe exifted in the abdominal vifcera, the abdomen was opened, but upon the moft careful infpection, no difeafed changes could be difcovered. The vifcera of the thorax were alfo all in a perfectly healthy ftate.

" The head was next examined.—The different finufes of the dura mater were larger than they are ufually found at this child's age, and they contained more than the common quantity of blood.

" The veffels of the pia-mater were unufually empty, but a ferous fluid had been effufed between the laminæ of which this membrane is compofed.

" When the fuperior part of the hemifphere of the brain was removed, the upper furface of the ventricles appeared more prominent than ufual; and when this part was ftruck, a general undulatory motion of the brain was produced.

" An opening was made into the right lateral ventricle, and five ounces of water were difcharged from the four ventricles, leaving the parts which formed them widely feparated.

" The membrane which furrounds thefe cavities exhibited no fign of inflammation, and no other marks of difeafe could be detected."

---

Hydrocephalus is a difeafe which, like many others, is fo clearly and forcibly defcribed in books, that one would fuppofe it might in all cafes be readily recognifed. It is the prominent features, however, alone that are thus laid down; the minuter fhades are difficult, and often impoffible to be well defcribed in words. The eye eafily fees what language is inadequate to paint. Hence it is, that a juft knowledge of the nature of difeafes is unattainable in any other way than by actual obfervation. But from the cafe above defcribed, and from others which I have witneffed, I am convinced that hydrocephalus is often prefent, without being marked by thofe ftriking appearances which are fuppofed effentially to characterife it. Thus the fymptoms which are laid down by the generality of writers as pathognomonic, as ftupor, dilatation of the pupils, preternatural flownefs of the pulfe, and convulfions, were none of them prefent, in any remarkable degree, in the cafe above related, till till towards the cloſe of the diſeaſe. At the ſame time, the reſemblance to common fever was ſo ſtrong, as to induce a hope, for a conſiderable time, that ſuch was the real nature of it. That hydrocephalus commences often with the common ſymptoms of fever, and none other, I am well convinced. In one caſe which I examined after death, I continued under this miſtake for the firſt week of the diſeaſe, when the nature of the complaint became evident, by the comatoſe and convulſive ſymptoms.

The moſt ſtriking circumſtances attending the above caſe were, the abſence of delirium and convulſion, the contractility of the pupil of the eye, and the regularity of the pulſe, which varied little from that of health for a long time, and was never intermittent or preternaturally ſlow. When theſe are preſent, together with the common febrile ſymptoms, it is not ſurpriſing that the nature of the diſeaſe ſhould be overlooked, and the moſt favourable moments for relieving it loſt.

Reſpecting the mode of treatment which was adopted, many, I have no doubt, will conſider it as inert and trifling. It was, no doubt, influenced by the uncertainty which exiſted reſpecting the nature of the diſorder. Whilſt a hope remained that ſimple fever was preſent in a mild form, going on to its natural termination or criſis, very free evacuations or very active means of any kind, were hardly admiſſible; eſpecially as the former were not indicated by any marks of active inflammation in the ſyſtem. But even if there had been leſs doubt of the nature of the diſeaſe, and the marks of oppreſſed brain had been more apparent, I am not ſure that I ſhould have purſued a very different mode of practicefrom that which was actually employed. The effects of mercurials, and the other remedies commonly recommended for hydrocephalus, afford little encouragement to imitate them. If recoveries have ſometimes followed their uſe, they have at leaſt as often failed. Their powers, therefore, are at beſt equivocal, and poſſibly they may ſometimes have done harm. Recoveries again have taken place where none, or comparatively inactive remedies, have been had recourſe to. A few months back I had under my care, in the Diſpenſary, a boy of nine years of age, of a ſcrophulous habit, ill-fed, with a pale and ſallow ſkin; in whom marks of irritated brain aroſe, but with ſome peculiarity of ſymptoms. The pulſe and breathing were in this caſe both exceedingly rapid, the tongue was furred, bowels coſtive, pupils exceedingly dilated, with great pain in the head, and confuſed viſion; there was likewiſe ſome diſorder of the intellect. Bliſters were applied to the temples, and as there was a perpetual reſtleſſneſs preſent, (he dozed and moaned frequently, but had no ſound ſleep), one drop of tincture of opium was given every two hours, and the feet were bathed in warm water. The bowels were opened by ſcammony and and calomel. By thefe means, in three or four days, the fymptoms gradually difappeared, the quicknefs of pulfe fubfided, and the pupils recovered their contractility. The fame fymptoms returned about a fortnight afterwards, and again yielded to the repetition of bliſters with opium.

The great fatality of hydrocephalus, in fpite of the moſt powerful modes of treatment, may be gathered from the following ſtatement, which I might eafily have enlarged. Mr. I. PAISLEY relates a cafe, in the *Ed. Med. Eſſ.* v. 3. p. 23, in which bliſtering, cupping, and fcarification of the head, with other remedies, were ineffectually employed. He mentions his having met with many fimilar cafes, which terminated fatally. Dr. PERCIVAL, in a letter to Dr. DUNCAN, containing mifcellaneous practical obfervations, has fome remarks on hydrocephalus internus. (*Med. Comment.* v. 5. p. 174).

"The fatality (he fays) of this difeafe has been acknowledged and lamented by the moſt experienced and intelligent phyficians. The late Dr. WHYTT, of Edinburgh, has recorded twenty cafes, which baffled all his ſkill and judgment; and a phyfician of the higheſt reputation, in his excellent remarks on this difeafe, candidly confeffes, that it is not in his power to fuggeſt any probable means of curing it; and that it has hitherto difappointed all his attempts, both when confided in alone, and in confultation with the ableſt of the faculty. (*Med. Obſ. & Inq.* v. 5. p. 40.")

Such was the fatality of the difeafe before mercury was fuggeſted for its cure by Dr. DOBSON; and we ſhall not find that it has been materially leffened fince. The cafes of fuccefs are few; thofe of failure are as numerous, and of thefe a few only have probably been recorded. Dr. S. F. SIMMONS, in a letter to Dr. Duncan (*Med. Com.* v. 5. p. 415), endeavours to ſhew that the good effects which have occafionally been obferved to follow the ufe of mercury in this difeafe, are more attributable to the bliſters, and other remedies, which have been employed in conjunction with it, than to the mercury itfelf. He remarks, that falivation by mercury is fometimes preceded by convulfions in very young fubjects. He was informed by Dr. ODIER, of Geneva, that the practitioners of that city, had often fucceeded by large bliſters to the head. Dr. DAWSON lays great ſtrefs on opiates in thefe cafes, for the purpofe of obviating fpafm. He advifes to cover the whole head with bliſters, and to apply them likewife behind the ears. In two cafes which terminated fuccefsfully, he afcribed the cure to the Theriaca Andromachi; but bliſters had been conjoined. Mr. WILMER, in his "*Cafes and Remarks*," records the unfuccefsful employment of mercury, though falivation was induced by it. Dr. EASON, of Mancheſter, cured a cafe of hydrocephalus by mercury, but it was of the chronic kind (*Med.*

*Com.*

*Com.* v. 8. p. 325). Dr. Aery, of Whitehaven, alfo fucceeded by the ufe of mercury in one inftance (*Ibid* 322). Dr. A. Hunter, of York, relates a cafe which was cured by the vapour-bath; but this alfo was of the chronic fpecies (*Ibid*). Dr. A. Campbell, of Hereford, fays: "In a long courfe of practice, I have attended many patients ill of hydrocephalus internus, and am forry to fay, I never knew more than one recover; a young man of twenty-five, who was recovered by repeated bleeding, and purging with falts." He then mentions two cafes in which mercury was employed; one of which was cured, the other terminated fatally (*Med. Com.* v. 9. p. 240*). A further inquiry into the event of cafes recorded, would afford an equally unfatisfactory refult with thofe above adduced.

From thefe, therefore, it is impoffible to place much reliance on any of the modes of treatment which have been adopted hitherto, or to be very fanguine in our expectations of relief from art. We have feen the difeafe combated by very different means; by bleeding, and other evacuants; by bliftering; by opium; and by mercury. Cures have fucceeded to all, but they have all as frequently failed. Others again have recovered, where no active means were employed. In the laft autumn, when the malignant fore-throat with fcarlet eruption, was very prevalent and fatal in and about London, I attended, with Dr. Saunders, a little girl about ten years of age, who, on the fubfidence of the febrile fymptoms, was attacked with anafarca. From the affection of the head, which now arofe, and which kept pace exactly with the effufion in the cellular membrane, I have no doubt that effufion had equally taken place in the brain. Conftant delirium was prefent, with little or no fever, and the pupils were greatly dilated. Violent convulfions alfo came on, and continued for many hours, when a purgative was given for the purpofe of producing abforption: this end was effected in fome degree, with proportional relief of the affection of the head; but a few hours again produced accumulation in the cellular membrane, and difturbance of the intellectual faculties. This change I obferved more than once, and it is perfectly analogous to what takes place in dropfies, in debilitated habits, where purgatives relieve for the inftant, but rather, by their weakening effects, favour further accumulation afterwards. It was determined, therefore, to leave the cafe to nature, in the expectation, that as the general ftrength returned, the extreme veffels would likewife recover their healthy action, and the fuperfluous fluid be removed. In this we were not deceived.

The

* Dr. Paterson found, on collecting the histories of hydrocephalus, in which mercury had been employed, that the number of patients who died was greater than those who recovered, under its administration, in the proportion of twenty-two to twenty. (*Vide* "Paterson's Letters to Quin, on the Internal Dropsy of the Brain." Dublin. 1794.)

The anafarca and the affections of the head both gradually difappeared together; the mental faculties, however, remained for fome time impaired, a degree of childifh nefsad fatuity fubfifting for feveral weeks.

Upon the whole, it is fair to conclude, that the recoveries which have actually taken place in this difeafe, are more owing to natural efforts, than to the interference of art. Hydrocephalus, like other difeafes, differs greatly at different times, in degree of violence and of danger. It arifes in fubjects very oppofite to each other, and therefore not likely to be benefited, exclufively, by any one mode of treatment. It would be well, however, if we knew more than we do of its intrinfic nature, or proximate caufe. From its accompanying general anafarca, as it often does, it is, in fuch cafes, probably founded in the fame general caufes, and calls for a fimilar mode of treatment. No one would think here of blood-letting, or other debilitating remedies. This, perhaps, is the fpecies of the difeafe in which bliftering promifes moft, and to which the ufe of mercury appears beft adapted, from the power which thefe remedies poffefs of promoting abforption. Some, as Drs. WITHERING, QUIN, and others, confider hydrocephalus as originating in inflammation; and the accumulation as a confequence only of this. Traces of inflammation have not commonly been obferved on diffection after death, though they fometimes have; yet there is little doubt that many cafes of the difeafe are accompanied with an increafed or inflammatory action of the veffels of the brain. This is evident from the head-ach, throbbing of the arteries, flufhing of the face, parched tongue, and other febrile fymptoms, which prefent themfelves. Blood-letting and the antiphlogiftic plan feem here to be particularly indicated, and have, no doubt, in fuch cafes been fuccefsful: where, however, the general fyftem partakes little of the inflammatory diathefis, and the pulfe is little irritated (and that there are fuch cafes, is proved by the one detailed above), I fear the effect of general evacuations will be lefs favourable. Hydrocephalus, too, moft frequently occurs in fcrophulous and rickety habits, which ill bear evacuations of any kind, to any extent. The tendency to difeafe, is in thefe cafes fo ftrong, and efforts of art in general fo unavailing, that recoveries cannot be expected often to occur. This affection is well known frequently to pervade families, affecting all, or the greater part of the children, at a certain age. This fhews how much it depends on general habit, rather than local affection; were it the refult only of occafional or accidental caufes, our efforts to relieve it would more frequently be attended with fuccefs.

Dr. Withering has fuggefted the employment of digitalis as a remedy in hydrocephalus; but it has not yet received the fanction of experience. To what particular ftate of the difeafe it may be adapted; whether it might relieve by diminiſhing arterial action, or by its power as a diuretic, on the ſame principle that it ſucceeds in the cure of dropſy, is uncertain, and only to be determined by cautious experiment.

Hydrocephalus is eſpecially liable to be overlooked in infancy, and to be paſſed by under the general, and, for the moſt part, unmeaning appellations of *teething* and *worms*. Theſe are convenient terms for the indolence of the practitioner, and ſerve to ſatisfy the anxious mind of the parent. I am acquainted with two inſtances where the caſes were thus lightly treated, and the danger overlooked, and that too by practitioners of ſome note, both of which terminated fatally in a few hours. Diſſection afterwards pointed out their real nature. If the nature of this diſeaſe be ſuch, as I fear it is, that it will in the majority of caſes prove fatal, in ſpite of any treatment, it is ſtill of importance that it ſhould be early ſeen, and not confounded with others of little, or inferior magnitude. Both the character of the phyſician, and the happineſs of families are implicated in this.

---

